# Stressors and coping strategies among physiotherapy students: Towards an integrated support structure

**DOI:** 10.4102/hsag.v23i0.1091

**Published:** 2018-08-16

**Authors:** Elizabeth C. Janse van Vuuren, Karen Bodenstein, Mariette Nel

**Affiliations:** 1 Department of Physiotherapy, University of the Free State, South Africa; 2 Department of Biostatistics, University of the Free State, South Africa

## Abstract

**Background:**

Stress is a major problem among university and, specifically, health care students, as it may influence academic performance and psychological well-being negatively.

**Aims:**

To develop and implement a student support system based on the perceived stress, stressors and coping strategies of physiotherapy students.

**Methods:**

A cross-sectional, descriptive study was undertaken, using a literature-based, self-compiled questionnaire and the 28-item General Health Questionnaire (GHQ-28). Over a period of three years, 207 third- and fourth-year physiotherapy students at a South African university were included.

**Results:**

Psychological distress was experienced by 61.8%–71.2% of participants. During the 3 months prior to the study, 6% of participants received psychological or psychiatric help and 9% of participants used some form of psychiatric medication. The main stressors identified during clinical training were the suffering and death of patients, academic pressure and tension during interaction with personnel. Participants indicated that they mainly coped with these stressors by talking to someone such as a family member or a friend.

**Conclusions:**

Based on the findings of this study, a framework to identify and support students in pre-clinical and clinical training years was developed and implemented over five years. This proposed framework might positively contribute to the psychological well-being of health care students.

## Introduction and theoretical background

Stress is a major problem among higher education students as they try to manage academic, social and personal challenges. A wide spectrum of factors associated with stress in this group of students has increasingly been researched in the last decade. Excessive or unconstructive stress negatively influences academic performance and health and can lead to psychological distress with symptoms of anxiety and depression. Continued psychological distress could cause academic burnout, a state of emotional exhaustion which results in students experiencing emotions of decreased personal achievement and disinterest in studies, further negatively impacting academic learning (Lin & Huang 2014; Tomaschewski-Barlem et al. 2013).

The stressors as well as the coping strategies, utilised by health care students, have also been well documented in recent years (Jacob, Itzchak & Raz 2013; Sabih, Siddiqui & Baber 2013; Sani et al. 2012; Walsh et al. 2010). Levels of stress in health care students have been reported to be so high that it could even be linked to psychological morbidity. Psychological morbidity is associated with a deviation from physical or psychological well-being and can be linked to an above threshold score on the General Health Questionnaire (GHQ) as developed by Goldberg et al. (1978). A study among undergraduate physiotherapy students revealed an above threshold score on the 12-item General Health Questionnaire (GHQ-12); thus connecting 27% of these students to probable psychological morbidity (Walsh et al. 2010). In another study among health care students (including physiotherapy), Sabih et al. (2013) found that 88% of the participants reported feeling stressed, with 40% being moderately stressed and 6% being severely stressed. Sreeramareddy et al. (2007) linked 20.9% of undergraduate medical students in their study to psychological morbidity utilising the same GHQ-12 questionnaire. Such high levels of stress have been linked to poor academic performance (Alika 2012) and even burnout (IsHak et al. 2013).

Several authors highlighted academically related factors as the major stressor in health care students, followed by other stressors such as personal, financial, psycho-social or emotional factors (Jacob et al. 2013; Tucker et al. 2006). Specific academically related factors were programme overload, severe time demands or time management problems, long hours of study resulting in limited free time as well as difficulty in balancing academic and personal lives (Alika 2012; Elias, Ping & Abdullah 2011; Jacob et al. 2013; Mohanty et al. 2011; Pereira & Barbosa 2013; Tucker et al. 2006; Walsh et al. 2010).

A further dimension of potential stress is added in health care programmes with the introduction of clinical placement. Clinical placement refers to the placement of a medical, nursing or allied health care student in a primary, secondary or community health care setting where they have to provide discipline-specific health care to patients. Singh, Sharma and Sharma (2011) reported that 67% of nursing students experienced moderate to severe stress levels, specifically during clinical practice. In accordance with this, Helmers et al. (cited in Mahajan 2010) found that medical students experienced increased levels of stress with the move from basic to clinical training. Contact with patients (Mahajan 2010) and communicating with the patients’ family or caregivers (Thomson et al. 2014) are perceived as major factors contributing to stress in undergraduate health care students. The interaction with clinical educators is another stressor during clinical training (Thomson et al. 2014).

In light of the numerous stressors among healthcare students, it is essential to consider the coping strategies utilised by these students in an effort to address their levels of stress. General coping strategies, alluded to in literature, include planning and prioritising, active coping, self-distraction or making time for leisure activities, avoidance, problem-solving and emotional support (Pereira & Barbosa 2013; Singh et al. 2011; Sreeramareddy et al. 2007). Pereira and Barbosa (2013) further suggest that students also cope better when they realise their own limits and do not compare themselves with fellow students. One of the main coping strategies of health care students is talking to someone. Participants in the study conducted by Powell and Toms (2014), indicated that they would rather confide in peers than senior staff members or clinical educators, as they felt that their peers understood their emotions, knew what they were going through and might have had similar experiences. This is especially applicable after a patient’s death when students often feel extremely guilty or incompetent and need to be reassured. Despite these coping strategies utilised by health care students, Thomson et al. (2014) still found that 6% of students in their study needed external support as a result of stress in clinical settings.

External support structures for university students are widely described in literature and can be divided into academic support (Dawson et al. 2014; Hoyne & McNaught 2013), emotional support (Regehr, Glancy & Pitts 2013) or an integration of academic and emotional support through mentoring (Gershenfeld 2014). Because of the nature of stressors experienced by health care students during clinical training, emotional support structures for these students are more frequently described in literature than academic support structures. This, however, does not imply that health care students do not need or could not benefit from academic support and mentoring. Hoyne and McNaught (2013) found that health care students with known academic difficulties were unwilling to engage with academic support structures, necessitating the implementation of compulsory academic support for students performing below a set benchmark. The authors of this article, however, found little evidence of an external support structure that integrates emotional support, academic support and mentoring as a comprehensive approach in managing stress among health care students.

As lecturers in physiotherapy, the authors observed emotional stress among students during clinical training (third and fourth study years) even though most students were academically successful. This became evident through the emotional vulnerability of students (e.g. tearfulness and crying), volatile relations between students and clinical educators as well as an increase in individual student consultations with lecturers. This situation raised questions with regard to the perceived stress and coping strategies among our students as well as the support provided by the department, as the existing support structure consisted of informal referral of isolated student cases as they presented. Even though vast literature is available on stressors and coping strategies among university and specifically health care students, limited information on these topics still exists for physiotherapy students and formed the basis of this study. For the purpose of this study, the researchers defined stressors as an event that caused stress in students, specifically during clinical training, coping strategies as specific efforts to minimise these stressful events and perceived stress as ‘caseness’ (Jackson 2007) or ‘distress’ (Sterling 2011) displayed through an above threshold score on the 28-item General Health Questionnaire (GHQ-28).

The specific research aims for this study were as follows:

- to determine the perceived stress among physiotherapy students by utilising the GHQ-28
- to determine the stressors and coping strategies employed by physiotherapy students by means of a self-compiled questionnaire
- to propose an integrated student support structure for physiotherapy students based on the identified stressors and coping strategies.

## Study design and methodology

**FIGURE 1:**
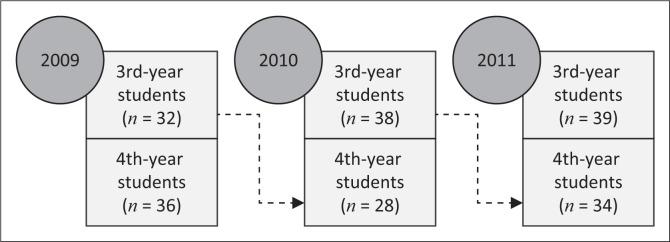
Student groupings that completed the questionnaires (*n* = 207).

The study, reported here, employed a cross-sectional, descriptive study design to determine the perceived stress, stressors and coping strategies among physiotherapy students, specifically during clinical training. The study population included all third- and fourth-year physiotherapy students at the University of the Free State (UFS) for three consecutive years (2009–2011). The study was repeated over a period of 3 years in order to limit the impact of incidental or year-group specific aspects and establish a reliable picture of perceived stress, stressors and coping strategies. The data therefore contained two groupings: (1) a group that repeated the questionnaires as third- and fourth-year students; and (2) a group that completed the questionnaires either in their third and fourth year of study (see Figure 1).

Two questionnaires were utilised for purposes of data collection: (1) the GHQ-28 questionnaire to determine the participants’ perceived stress; and (2) a literature-based, self-compiled questionnaire to obtain basic sociodemographic information and information on stressors and coping strategies during clinical training.

The GHQ-28 is a self-assessment questionnaire consisting of four sub-scales, containing seven items each, measured on a four-point Likert scale. These sub-scales are (1) somatic symptoms; (2) anxiety and insomnia; (3) social dysfunction; and (4) severe depression. The GHQ-28 measures the general level of psychological health of the individual (Sterling 2011). This 28-item version of the GHQ is the most frequently used and therefore allows for more valid comparisons (Jackson 2007). In this study, results from the GHQ-28 were analysed, utilising the binary coding and scoring method. With this scoring method, participants’ total score (i.e. the sum of the sub-scales’ scores) on the GHQ-28 is determined and, if the total score exceeds 4, it indicates ‘caseness’ (Jackson 2007) or ‘distress’ (Sterling 2011).

The reliability coefficient for the GHQ ranges between 0.78 and 0.95 in various studies worldwide (Jackson 2007). In the South African context, similar reliability coefficients have been reported, namely a 0.91 by Bosman (in Koen et al. 2011), 0.84 for the total scale among a group of professional nurses by Koen et al. (2011) and ranges between 0.70 and 0.83 (for all sub-scales) among a black South African sample (De Kock, Görgens-Ekermans & Dhladhla 2014). In the same study, the GHQ was also shown to be valid (*pclose fit* = 1.00) for this South African sample (De Kock et al. 2014).

For enhancement of data as well as purposes of triangulation, a self-compiled questionnaire was utilised for the collection of basic demographic information (mostly by means of closed-ended questions) and data on stressors, and coping strategies (mostly by means of open-ended questions). Examples of the open-ended questions are as follows:

- Which aspects or incidents during clinical training upset or worry you?
- How do you handle the above-mentioned aspects or incidents?

The open-ended questions were coded separately by assigning a code to each of the answers provided by the participants. Initially, similar answers were coded with the same code. After the initial coding, the answers were read and re-read in order to identify any additional correlating codes, which were then grouped together. The final codes were reported on quantitatively and utilised for a more accurate description of the stressors and coping strategies of the participants in this study.

A pilot study was conducted among 10 occupational therapy students to test the study procedure as well as the applicability and clarity of questions included in the questionnaires. No changes were made to the data collection tools following the pilot study. Data collection in the main study was conducted upon completion of clinical training for each year in order to provide participants with an opportunity to reflect on their total clinical experience. Questionnaires were completed in an examination room setting to prevent discussion of questions and contamination of results. A psychologist was present during all data collection sessions in order to immediately address any emotional reactions elicited by the questionnaires. Questionnaires were numbered to ensure confidentiality, but students could assign a pseudonym to their questionnaires. By means of this pseudonym, they could acquire access to their specific results after completion of the study.

Descriptive statistics were calculated, using frequencies and percentages for categorical data, and medians and percentiles for continuous data per group. The groups were compared by means of 95% confidence intervals for unpaired data.

## Ethical considerations

Ethical clearance was obtained from the Ethics Committee of UFS, Faculty of Health Sciences (ECUFS number 71/09), before the commencement of the study. Informed consent was obtained from all study participants.

## Results

### Demographic information

For the three years, the participants’ ages ranged between 20 and 32 years, with the median age of third-year students being 21 years and fourth-year students being 22 years. The distribution of gender among participants was 29% male and 71% female.

### Perceived stress, stressors and coping strategies during clinical training

**FIGURE 2:**
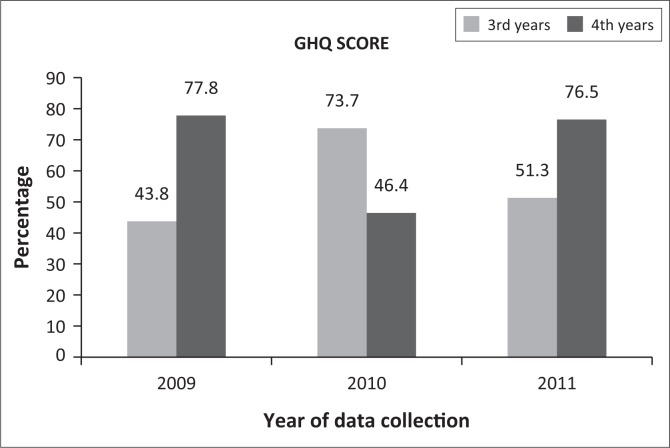
Psychological ‘caseness’ or ‘distress’ based on the GHQ score for third- and fourth-year students (2009–2011).

Perceived psychological well-being was reported by the participants’ total score on the GHQ-28. Results for psychological ‘caseness’ (Jackson 2007) or ‘distress’ (Sterling 2011) of third- and fourth-year physiotherapy students are displayed in Figure 2.

Psychological ‘caseness’ or ‘distress’ on average was 61.8% in 2009, 71.2% in 2010 and 63% in 2011, with the highest percentage being 78% among fourth-year students in 2009. Results from the open-ended questionnaire indicated that over the period of three years, a total of 12 participants (5.8%) had received psychological or psychiatric help in the three months prior to completing the questionnaire and 17 participants (8.2%) had used some form of psychiatric medication. Of those participants that had received psychological or psychiatric help, 36% (*n* = 12) indicated that clinical work and/or their studies were the reason for seeking help. No tendencies or associations could be found between the GHQ total scores and results on stressors and coping strategies (see Table 1), although tendencies were observed for receiving psychiatric help and using psychiatric medication in the past three months.

**TABLE 1:** Comparing stressors and coping strategies with GHQ total scores.

Variables	Year	Yes		No		95*%* Cl for *%* difference
		*%*	*n*	*%*	*n*	
Received psychiatric help in the past three months	2009	40	2	63.5	40	−53.7; 15.4
	2010	100	1	70.8	46	−50.7; 41.2
	2011	83.3	6	60.6	40	−18.4; 40.9
Used psychiatric medication in the past three months	2009	80	4	59.7	37	−23.6; 40.9
	2010	71.4	5	72.7	40	−38.2; 22.8
	2011	100	5	60.9	39	−5.8; 51.3
Discuss problems with someone	2009	61.5	40	66.7	2	−34.9; 42.0
	2010	71.9	46	100	1	−40.1; 51.8
	2011	62.1	41	66.7	4	−31.1; 33.7
Participate in outdoor activities	2009	60.3	35	70	7	−32.8; 22.8
	2010	69.5	41	85.7	7	−33.5; 22.2
	2011	61.9	39	70	10	−30.9; 24.2

Note: Comparing the ‘yes’ with ‘no’ responses for all students (third and fourth years).

CI, confidence interval.

A variety of stressors were identified by the participants, but, interestingly, the main stressors for both year groups were similar, namely suffering and death, academic pressure and tension during interaction with qualified personnel. The suffering and death of patients during clinical training was the stressor that scored the highest for both third- and fourth-year students in all the years of data collection (see Table 2). Academic pressure and tension during interaction with qualified personnel (i.e. communication, referral and interaction with all members of the multi-disciplinary team, including interaction with the qualified physiotherapists and academic staff) had more comparable scores, but noticeably less than the suffering and death of patients.

**TABLE 2:** Stressors for third- and fourth-year groups during clinical training.

Variables	Suffering and death (%)	Academic pressure (%)	Tension during interaction with personnel (%)
**Third-year (*n* = 109)**			
2009 (*n* = 32)	59	25	25
2010 (*n* = 38)	58	37	16
2011 (*n* = 39)	59	18	10
**Fourth-year (*n* = 98)**			
2009 (*n* = 36)	39	25	33
2010 (*n* = 28)	71	18	39
2011 (*n* = 34)	59	35	29

Note: Values in italics highlight the significance of the finding that in all groups for all three years, suffering and death were the most important stressor.

Table 3 indicates that participants in both the third and fourth study years mostly coped with these stressors during clinical training by *talking to someone* such as a family member or a friend (varying between 39% and 71%). There is less reliance on crying (3%–13%), with avoidance being a coping mechanism for between 11% and 21% of the participants. Again similarity in the patterns of coping between third-year and fourth-year students existed. In addition, a high percentage of participants (87%) mentioned in the open-ended questions that they participated in outdoor and recreational activities even though they did not perceive and indicate this as a main coping strategy.

**TABLE 3:** Main coping strategies for third- and fourth-year groups during clinical training.

Variables	Talk to someone (%)	Cry (%)	Avoidance (%)
**Third-year (*n* = 109)**			
2009 (*n* = 32)	63	13	16
2010 (*n* = 38)	68	3	11
2011 (*n* = 39)	59	8	21
**Fourth-year (*n* = 98)**			
2009 (*n* = 36)	39	6	14
2010 (*n* = 28)	71	7	14
2011 (*n* = 34)	62	9	12

Note: Values in italics highlight the significance of the finding that in both groups for all three years, talking to someone was the most important coping strategy used.

## Discussion

Regarding our first two research aims of exploring the perceived stress as well as stressors and coping strategies among physiotherapy students at our institution, the participants demonstrated a high level of perceived stress (more than 44% of participants over all three years of data collection) according to the GHQ-28. This percentage is high compared to other studies utilising by the GHQ, in which only 27% of physiotherapy students (Walsh et al. 2010) and 21% of undergraduate medical students (Sreeramareddy et al. 2007) presented with psychological morbidity. A study among dental students, however, revealed that 53% of students are presented with psychiatric morbidity (Makhal et al. 2015) which concurs with university students in Iran where 49% (Mokhtari et al. 2013) were classified with mental disorders. The lowest percentage of psychiatric caseness (17%) was found among students in the United Kingdom which correlates with that of the general population in the United Kingdom, namely 18% (Macaskill 2013). Because of the high stress experienced by health care students, Sandars et al. (2014) suggested that student support should be an ‘integral’ part in health education and made available to all students and not only to those who ‘struggle’.

The high level of perceived stress of participants at our institution supported our third research aim of proposing an integrated support structure for physiotherapy students. From the data obtained during our study as well as relevant literature, we identified four key directives for the development of a potentially effective integrated student support structure.

The first and most important directive relates to the need for the integration of emotional and academic support within one support structure, as existing structures, described in literature, focused only on one of these aspects (Dawson et al. 2014; Hoyne & McNaught 2013; Regehr et al. 2013). Both academic and emotional aspects were noted as major stressors in our study (see Table 2), indicating the integration of these aspects within a student’s frame of reference. On the emotional side, the suffering and death experienced by patients was the most important stressor for students in our study. This concurs with the findings of Laranjeira (2011) as well as those of Jan and Popescu (2014) who found that students experienced stress as a result of patient suffering and death. Opportunities to address these types of emotional stressors might be created through debriefing sessions (Powell & Toms 2014) as well as mentoring programmes (Gershenfeld 2014). Gershenfeld (2014) also described peer mentoring as an option to integrate the academic and emotional support for students. Pure academic stress, on the other hand, may be addressed by peer tutoring. Dawson et al. (2014) describe how tutoring supports student success, student retention and ultimately student graduation rates.

The second directive is based on creating appropriate communication platforms on different levels, as communication was an aspect included both under stressors (see Table 2) and coping strategies (see Table 3) in our study. The opportunity to *talk to someone* as a major coping strategy, as found in this study, is similar to the findings of many other studies in this field (Fornés-Vives et al. 2016; Jan & Popescu 2014; Jensen et al. 2016; Laranjeira 2011; Wolf, Stidhamb & Ross 2015; Yamashita, Saito & Takao 2012). Patient suffering and death as well as tension during interaction with qualified personnel were indicated as two major stressors in our study (see Table 2). Powell and Toms (2014) link these two aspects when they refer to the intensified stress experienced by students because of qualified personnel’s (undesired) reactions following patient deaths. This emphasises the importance of good communication platforms and the necessity of qualified personnel being positive role models and creating an effective learning environment (Powell & Toms 2014).

The third directive addresses the availability of support in both pre-clinical and clinical years. As reported in other studies (Divaris et al. 2013; Mokhtari et al. 2013; Makhal et al. 2015), academic factors were linked to stress experienced by students in this study (see Table 2). Academic support, however, should be offered from the pre-clinical years, and this is supported by Hoyne and McNaught (2013) who indicated that academic support courses should be compulsory, especially for health care students because of their reluctance to voluntarily attend such sessions. Esa et al. (2010) also identified an increased level of academic stress among university students because of a lack of soft skills which might be addressed by academic support courses in early study years. Students entering university, however, also have to make a number of adjustments. Personal psycho-social and emotional factors associated with these adjustments have been listed as contributors to stress and include high expectations of parents and teachers, high self-expectations, competitiveness, inadequate accommodation, interpersonal or family problems, sleeping problems as well as health problems (Alika 2012; Chilukuri et al. 2012; Pereira & Barbosa 2013; Sani et al. 2012; Sreeramareddy et al. 2007).

The fourth directive relates to the much needed interface between student support actions and external referral or support. The importance of external referral is confirmed by some participants in this study who received psychological or psychiatric help during the three months prior to the study and 17 participants (9%) using some form of psychiatric medication, similar to the 5% receiving treatment for psychological problems as found by Macaskill (2013). Thomson et al. (2014) also found that a small number of students needed external support in their study as a result of stress in clinical settings.

Guided by the four directives discussed above, the development of an integrated student support structure in the Department of Physiotherapy at the UFS was a process over five years.

## Towards an integrated student support structure

**FIGURE 3:**
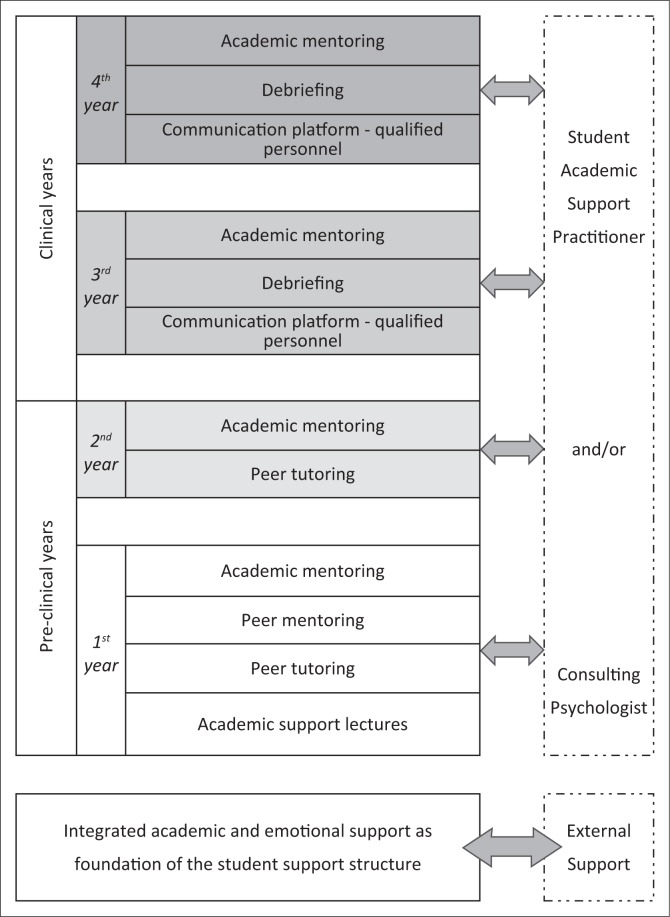
Integrated student support structure.

The proposed integrated student support structure, comprises of an integration of academic and emotional support was made available over all four study years (i.e. pre-clinical and clinical years) for Physiotherapy students. Figure 3 provides a visual representation of the integrated student support structure which is subsequently described in more detail.

Support in the first study year is equally distributed between academic and emotional support because of the academic and emotional adjustments students entering university must make. Academic support is provided through a peer tutoring system which entails face-to-face, small-group sessions facilitated by final-year physiotherapy students. During these sessions, the tutors support the first-year students academically by explaining difficult concepts and discussing previous assessments and/or examples of assessments. During 2012, additional academic support was implemented for first-year students by means of a series of institutional, compulsory academic support lectures. These lectures include the use of technology at university, prioritising and goal-setting, memory and information management, decision-making skills, motivation, creative and critical thinking as well as academic literacy as supported by Hoyne and McNaught (2013), and Esa et al. (2010). In 2017, orientation lectures by the Student Academic Support Practitioner were implemented. These lectures focus on study methods, test and examination techniques, time management and self-reflection techniques that students can utilise after assessment to identify challenging areas and improve future performance.

Academic support for first-year students is further enhanced by a dual mentoring system (where mentoring is provided by peers as well as academic staff) which aims at integrating emotional and academic support. Peer mentoring was previously provided by final-year physiotherapy students, but, with the development of this integrated student support structure, it was realised that senior students could not provide adequate emotional support to first years, as the focus of students changes from the pre-clinical to clinical years, influencing the mentorship relationship. The system thus was adapted in 2016. Peer mentoring currently is provided by second-year physiotherapy students who also still are in their pre-clinical study years. In addition to this peer mentoring system, academic mentoring is also provided to students. In the previous, more informal student support structure, academic mentoring was provided by the class guardian (academic staff member) of each year-group as needed. This meant that the academic mentor changed annually as the students moved through the different academic years. This was identified as a problematic area, as we realised that a more consistent mentorship relationship was needed. To address this need, an academic mentor was appointed in a full-time position in the department during 2014 (see Figure 3).

In the second study year, most students usually have successfully adapted to the transition to university and the support is therefore reduced. Only peer tutoring and academic mentoring are maintained and provided in the same way as during the first study year (see Figure 3).

With the transition from the pre-clinical study years to the clinical years (third- and fourth-year), the emphasis shifts towards emotional support because of the impact clinical work has on health care students (see Introduction). Differentiated support for students during clinical years is highlighted by Vogan et al. (2014) who links the additional requirements of these students to increased pressures and demands in the clinical environment. Firstly, a structured communication platform, which includes a two-way feedback-feedforward system involving students, academic staff members and qualified personnel, was established (see Figure 3). Academic staff members form the central link in this communication structure. Planned quarterly meetings take place between academic staff members and qualified personnel, whereas academic staff are constantly in conversation with students as part of clinical and academic training. This structure was further enhanced by the appointment of an academic mentor in 2014 who now creates a more centralised link among students, academic staff and qualified personnel. This academic mentor also ensures better integration (and a link) between academic and emotional support because of her centralised function.

In 2015, a continuation of high levels of perceived stress among students necessitated additional emotional support, and a formal debriefing system for students in their clinical years (i.e. third and fourth study year) was introduced (see Figure 3). These sessions are facilitated by academic staff members and relate to suggestions by Powell and Toms (2014) for the use of routine debriefing sessions for physiotherapy students during their clinical study years. These debriefing sessions provide students with the opportunity to *talk to someone* which was indicated in this study as the main coping strategy to handle stressors (see Table 3). In many cases, stress during clinical exposure is because of the death and suffering of patients (see Table 2), and these debriefing sessions appear valuable in addressing this aspect. Debriefing sessions furthermore provide valuable information to inform discussions during communications among students, academic staff members and qualified personnel (communication platform).

If problems, either academically or emotionally, persist following these interventions, external referral is facilitated. Within the integrated student support structure, external support does not operate in isolation, but forms part of the overall structure through effective feedforward–feedback communication channels. The importance and integration of external referral in a student support structure is confirmed by the finding that some participants in this study received psychological or psychiatric help during the three months prior to the study. In the proposed integrated student support structure, external referral is facilitated by the academic mentor and may include individual sessions with the Student Academic Support Practitioner or a consulting psychologist (see Figure 3).

Limitations of this study include the small sample size as well as the fact that continuous screening of students within all study years is not currently included as part of the support structure. An additional limitation is recognised, namely that variables such as past trauma, mood disorders and temperament, were not considered in this study.

A follow-up study with a larger study sample, including physiotherapy students from other universities in South Africa, could establish whether stressors experienced and coping strategies employed are universal or specific to a certain population. The study population could also be expanded to identify the stressors and coping strategies of the first- and second-year students (in their pre-clinical years) to determine the relevance and efficacy of the proposed student support structure to also provide optimal support during these study years. Future research should include continuous screening as well as reporting on other variables that could influence perceived stress among all students. This is necessary to evaluate the proposed student support structure and provide evidence on the effectiveness of the implemented interventions in order to continuously adapt the student support structure.

## Conclusion

The psychological distress of students continues to be high, and raises questions with regard to the effectiveness of existing programmes, probably because of a lack of the integration of academic and emotional support. Engagement of students with support programmes is also poor, possibly because of students not identifying their own support needs – a lack in relevance of more general support programmes or limited adaptation of programmes to meet the specific expectations of students. The implementation and constant refinement of support structures are therefore essential in higher education institutions and, specifically, health care programmes. The refinement of the proposed integrated support structure added valuable components such as a full-time academic mentor and debriefing sessions which combined academic and emotional support to students. Such an integrated structure, which provides academic and emotional support through both the pre-clinical and clinical study years, may be considered by universities offering health care programmes.
